# Non-Asbestos-Related Malignant Peritoneal Mesothelioma: A Diagnostic Challenge in a Case of Unexplained Ascites

**DOI:** 10.7759/cureus.95421

**Published:** 2025-10-26

**Authors:** Mohammad Armaghan Farooq Dar, Nyla Nasir, Ghulam Mujtaba, Muhammad Afzal

**Affiliations:** 1 Department of Gastroenterology, Royal Oldham Hospital, Oldham, GBR; 2 Department of Histopathology, Royal Oldham Hospital, Oldham, GBR

**Keywords:** ascites, case report, diagnostic challenge, immunohistochemistry, intraductal papillary mucinous neoplasm, malignant peritoneal mesothelioma, non-asbestos exposure, peritoneal biopsy, rare abdominal malignancy, unexplained ascites

## Abstract

Malignant peritoneal mesothelioma (MPM) is a rare and aggressive malignancy arising from mesothelial cells of the peritoneum. It is most frequently linked to asbestos exposure, although up to half of cases occur without such history. Because of its vague and non-specific presentation, diagnosis is often delayed; cytology is frequently non-diagnostic, and histological confirmation with immunohistochemistry is required. Prognosis remains poor, although advances in cytoreductive surgery and intraperitoneal chemotherapy have improved outcomes in selected patients. We describe a man in his late 60s with no history of asbestos exposure who presented with progressive abdominal distension over 18 months. He underwent repeated paracenteses with negative cytology and multiple imaging studies, initially attributed to an intraductal papillary mucinous neoplasm (IPMN) under surveillance. Subsequent computed tomography (CT) demonstrated extensive peritoneal disease with omental caking and splenic lesions. Omental biopsy confirmed epithelioid-type MPM with BRCA1-associated protein 1 (BAP1) loss on immunohistochemistry. Given advanced disease and poor performance status, the patient was deemed unsuitable for surgery or chemotherapy and was managed with best supportive palliative care. This case highlights the diagnostic challenges of MPM, particularly in the absence of asbestos exposure and in the presence of coexistent pathology, and underscores the importance of early histological evaluation in patients with unexplained recurrent ascites.

## Introduction

Mesothelioma is a rare and incurable malignancy associated with a poor prognosis. In the United Kingdom, approximately 2,700 new cases are diagnosed annually, while an estimated 30,870 cases were reported globally in 2020 [1]. Mesothelioma arises from mesothelial cells lining the serosal membranes, most commonly the pleura, where it is strongly linked to asbestos exposure. Recent studies have provided important insights into the molecular and clinical characteristics of malignant peritoneal mesothelioma (MPM) and highlighted the challenges associated with its early diagnosis and management [2].

MPM comprises three histological subtypes: epithelioid, sarcomatoid, and biphasic (mixed). Of these, the epithelioid subtype is the least aggressive and is associated with the most favourable prognosis [3].

The disease is strongly associated with occupational asbestos exposure and, less frequently, with other agents such as silica and radiation [4-7]. Clinical features are often non-specific, with patients typically presenting with abdominal distension, pain, nausea, anorexia, and weight loss [2,6]. Imaging may demonstrate ascites, omental caking, peritoneal thickening, and lymphadenopathy, and can also help distinguish MPM from peritoneal carcinomatosis through characteristic patterns of involvement [8-10].

A definitive diagnosis requires histological examination with immunohistochemistry [2,3]. Recent literature emphasises the importance of a multidisciplinary approach to MPM and highlights emerging therapeutic strategies, reflecting growing awareness of its rarity, diagnostic challenges, and aggressive clinical course [2,11].

Diagnosing MPM remains challenging because of its vague and overlapping clinical features, which often mimic benign or secondary peritoneal processes. Early symptoms such as abdominal distension, weight loss, and fatigue are frequently misattributed to more common gastrointestinal or hepatic disorders, leading to delayed recognition. Advances in molecular pathology, particularly the identification of BRCA1-associated protein 1 (BAP1) loss, have enhanced diagnostic precision and helped distinguish MPM from other peritoneal malignancies.

## Case presentation

We present the case of a 69-year-old man, initially reviewed by the surgical team for an inguinal hernia, with a past medical history of hypertension and diabetes. He was also under surveillance by the pancreatobiliary team for an intraductal papillary mucinous neoplasm (IPMN) diagnosed on imaging. He had no occupational asbestos exposure, having worked in the information technology sector.

His symptoms began with progressive abdominal distension over 18 months, leading to longstanding ascites of uncertain origin. He reported anorexia, weight loss, and fatigue, but no specific upper or lower gastrointestinal symptoms. He was followed up by the gastroenterology team for recurrent ascites refractory to medical management. A contrast-enhanced computed tomography (CT) scan of the abdomen demonstrated a large volume of non-dependent ascites, initially thought to be related to the IPMN.

The following table and subsequent figures illustrate key diagnostic and histopathological findings, each referenced in sequence within the text. Blood and ascitic fluid investigations revealed hypoalbuminemia (serum albumin 23 g/L) with ascitic albumin of 19 g/L, giving a serum-ascites albumin gradient (SAAG) of 4, suggestive of a non-portal hypertensive cause, possibly malignant. Ascitic fluid analysis showed high protein (45 g/L) and elevated lactate dehydrogenase (LDH). Key laboratory findings are summarised in Table 1, which demonstrates an exudative ascitic profile, while tumour marker alpha-fetoprotein (AFP), carbohydrate antigen 19-9 (CA19-9), and carcinoembryonic antigen (CEA) were within normal limits. Magnetic resonance cholangiopancreatography (MRCP) showed a stable uncinate IPMN without suspicious features, low-volume free fluid, and otherwise unremarkable abdominal viscera, including the liver, gallbladder, adrenal glands, and kidneys. The case was discussed at the regional pancreatobiliary multidisciplinary team (MDT) meeting, which considered multifocal branch-duct (BD) IPMN with the largest uncinate cyst measuring 50 mm, and recommended surveillance according to BD-IPMN guidelines.

**Table 1:** Laboratory findings with reference ranges. Values represent key blood and ascitic fluid investigations at presentation. LDH: lactate dehydrogenase; SAAG: serum-ascites albumin gradient; AFP: alpha-fetoprotein; CA19-9: carbohydrate antigen 19-9; CEA: carcinoembryonic antigen.

Test	Result	Reference range
Serum albumin	23 g/L	35–50 g/L
Ascitic albumin	19 g/L	–
Serum-ascites albumin gradient (SAAG)	4 g/L	>11 g/L = portal hypertensive; <11 g/L = non-portal
Ascitic protein	45 g/L	<25 g/L (transudate), >25 g/L (exudate)
Ascitic lactate dehydrogenase (LDH)	315 U/L	<200 U/L (commonly used cutoff; interpretation may vary with laboratory standards)
Alpha-fetoprotein (AFP)	<1.1 ng/mL	<10 ng/mL
Carbohydrate antigen 19-9 (CA19-9)	<1 U/mL	<37 U/mL
Carcinoembryonic antigen (CEA)	2 ng/mL	<5 ng/mL

**Figure 1:**
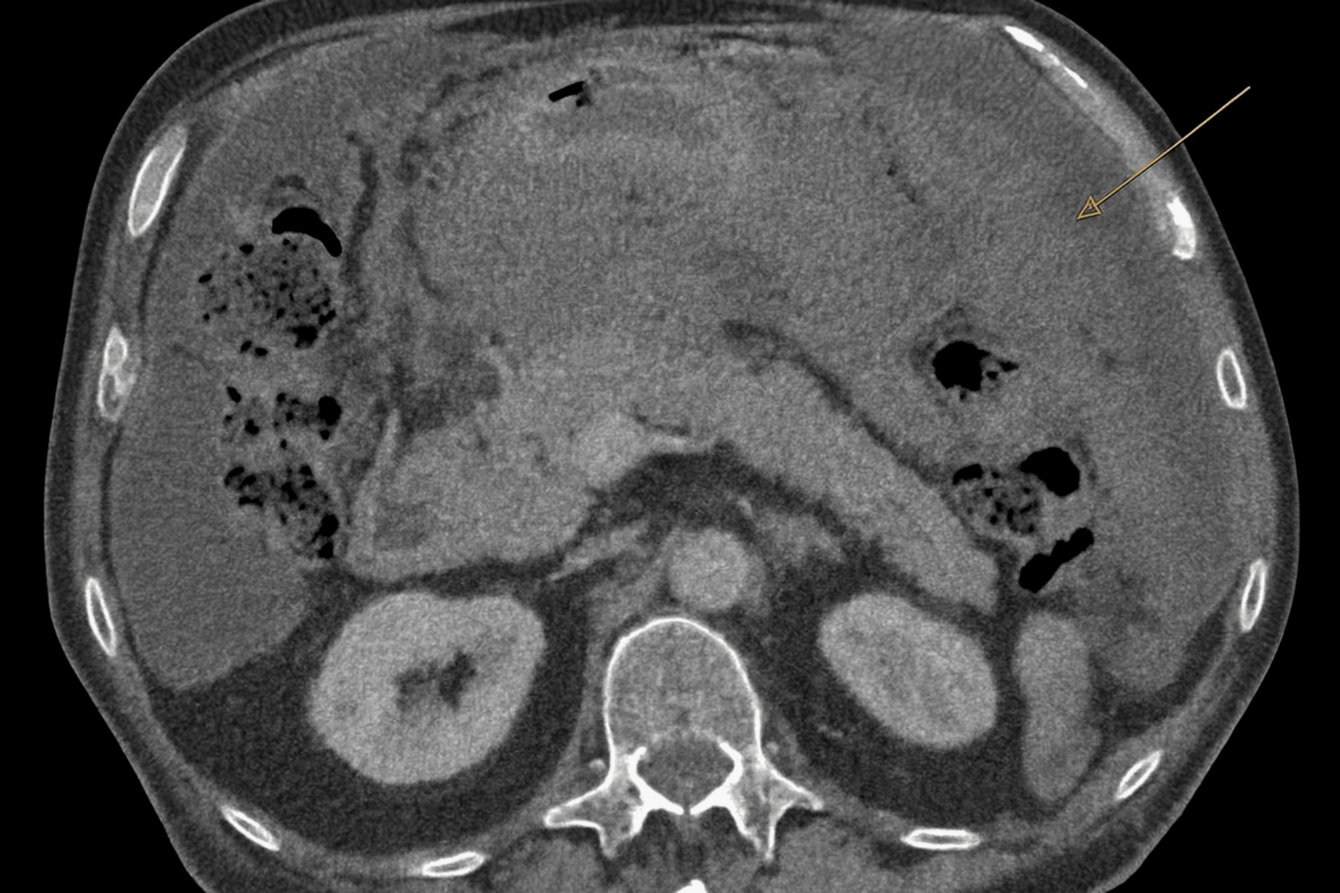
Axial computed tomography (CT) of the abdomen (portal venous phase) showing omental thickening and ascites. Axial computed tomography image of the abdomen (portal venous phase) demonstrates a sheet of omental thickening in the left hypochondriac region (arrow) with associated moderate ascites.

Despite multiple ascitic fluid samples sent for cytology, culture, and tuberculosis testing, no diagnosis was established. Serum tumour markers were within normal limits. His symptoms progressed, with worsening ascites. During an acute admission, he presented with abdominal pain and was assessed for a possible strangulated hernia. CT of the abdomen and pelvis demonstrated extensive peritoneal disease with omental caking, right inguinal hernia deposits, and new splenic lesions, consistent with metastases (Figure 1). A CT thorax showed left internal mammary and pericardial lymphadenopathy, without evidence of pleural disease. Following referral to the cancer of unknown primary MDT, an ultrasound-guided biopsy of omental deposits was performed.

**Figure 2:**
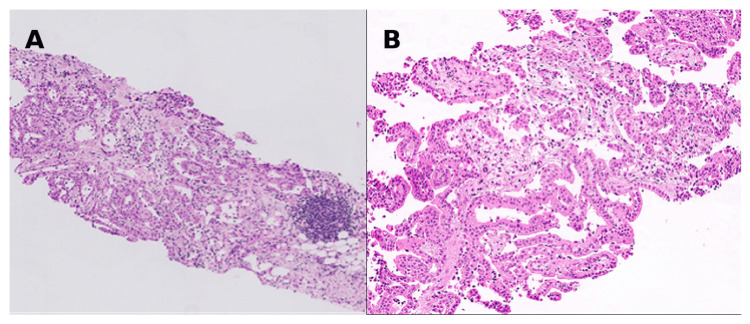
Core biopsy of the omentum (haematoxylin and eosin, H&E). Core biopsy of the omentum showing infiltration of fibroadipose tissue by an atypical mesothelial cell proliferation in the form of papillae and cystic glandular structures. The mesothelial cells are polygonal, with abundant, dense, eosinophilic cytoplasm and nuclei displaying prominent nucleoli. There is marked cellular atypia with nuclear pleomorphism. No evidence of necrosis is seen. (A) Haematoxylin and eosin, low power. (B) Haematoxylin and eosin, high power.

**Figure 3:**
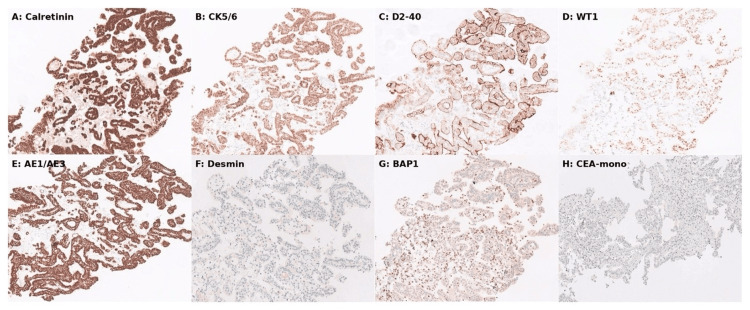
Immunohistochemistry panel of tumour cells. On immunohistochemistry, tumour cells show strong diffuse positivity for mesothelial markers, including calretinin (A), cytokeratin 5/6 (B), D2-40 (C), and Wilms tumour 1 (WT1) (D). AE1/AE3 (E) demonstrates strong diffuse cytoplasmic staining with perinuclear accentuation. The tumour cells are negative for desmin (F). There is complete loss of nuclear staining for BRCA1-associated protein 1 (BAP1) (G), consistent with BAP1 inactivation. The cells are negative for the epithelial marker carcinoembryonic antigen (CEA)-mono (H), excluding adenocarcinoma.

Histological examination of the biopsy specimen (Figure 2) showed cores of fibroadipose tissue infiltrated by an atypical mesothelial proliferation forming papillary and cystic glandular structures. The cells were polygonal with abundant eosinophilic cytoplasm and nuclei with prominent nucleoli. Atypical features included cellular and nuclear pleomorphism with scattered mitoses. Necrosis was not identified. Immunohistochemistry confirmed the mesothelial origin (Figure 3): tumour cells showed strong diffuse positivity for calretinin, CK5/6, WT1, and D2-40. AE1/AE3 demonstrated intense diffuse cytoplasmic staining with perinuclear accentuation. The tumour cells were negative for desmin. Nuclear staining for BAP1 was absent, consistent with BAP1 loss. The cells were also negative for epithelial markers, including CEA and Ber-EP4, excluding adenocarcinoma. The combination of epithelioid morphology, architectural and cytological atypia, invasion into fat, and loss of BAP1 supported a diagnosis of malignant mesothelioma of epithelioid type.

The case was subsequently discussed at the peritoneal tumour service MDT. Given disease progression and declining performance status, the patient was deemed unsuitable for chemotherapy, and best supportive palliative care was recommended.

## Discussion

Malignant peritoneal mesothelioma (MPM) is a malignancy arising from the mesothelial cells of the serosal membranes, most commonly seen in the pleura. The second most common site is the peritoneum, accounting for 10-20% of all mesotheliomas [2,8,9,11]. Mesothelioma has been linked to industrial mineral exposure, most commonly asbestos, with approximately 80% of cases related to asbestos exposure. While the association between asbestos and pleural mesothelioma is well defined, approximately 50% of cases of MPM occur without prior asbestos exposure [4-7,11], as in our case.

Other causative agents associated with MPM include therapeutic radiation for other malignancies [1,12-14] and exposure to radioactive thorium dioxide (Thorotrast), which was previously used as a diagnostic radiological contrast medium [4,6,11]. In certain geographic regions, including Turkey and North America, mineral fibres, such as erionite, fluoro-edenite, and possibly balangeroite, have also been implicated. Erionite, a silicate fibre, has been linked to an increased risk of pleural mesothelioma in Cappadocia, Turkey, where no asbestos exposure was identified [1,4,6]. Rare cases are caused by germline mutations of the BRCA1-associated protein 1 (BAP1) gene as part of the BAP1 tumour predisposition syndrome [2,5,13]. The role of other suspected risk factors in humans, such as simian virus 40 infection, remains unclear [4-6,15]. Mesotheliomas not attributable to asbestos exposure or any other known agent are considered rare [5].

Although the link between asbestos and MPM is less intense than with pleural mesothelioma, the proposed pathogenesis is similar. The deposition of small fibre particles or other chronic irritants damages DNA and induces the release of reactive oxygen species, resulting in genomic instability. The reactive oxygen and nitrogen released by mesothelial cells stimulate an inflammatory response by recruiting different molecules and proteins to enhance cell proliferation. Over time, this persistent chronic inflammation stimulates the carcinogenesis process of the mesothelial cells [2,5]. The interaction between environmental carcinogenic factors and genetic susceptibility may also play a role [2,13].

The typical growth pattern of MPM is insidious, with spread along the peritoneal surface and usually no involvement of abdominal organs or metastatic spread beyond the abdominal cavity [2,6,13-14,16]. Presenting symptoms vary but most commonly include generalised abdominal pain and distension due to ascites [2,6,11]. Occasionally, patients may present with a palpable abdominal mass [2,16]. Other non-specific symptoms include bloating, anorexia, weight loss, early satiety, and altered bowel habits [6,11,16]. An acute presentation may occur due to intestinal obstruction or perforation [2,6,13]. In some cases, the disease may be discovered incidentally on abdominal imaging or during surgery for another abdominal pathology [6,14], as in our patient who presented with a strangulated hernia.

Because of its rarity, insidious growth pattern, and non-specific symptoms, MPM is usually diagnosed late, at an advanced stage, when disease is already extensive [14], as in our case. The non-specific clinical findings of MPM contribute to delayed diagnosis; the average time of symptom onset to histological confirmation usually ranges from four to six months but may be longer [2,11]. By the time a diagnosis is established, most patients present with diffuse disease throughout the abdomen and have a poor prognosis [16].

Radiological findings are important in evaluating ascites of unknown origin. Contrast-enhanced computed tomography (CT) of the abdomen is the accepted first-line modality [13,16]. MPM typically appears as a heterogeneous soft-tissue mass with irregular margins that enhance with contrast. The pattern of spread is more expansive than infiltrative, with diffuse peritoneal thickening and omental caking [2,7,10,14,16]. A characteristic feature of MPM is its confinement to the abdominal cavity; extra-abdominal spread is rare and typically occurs via direct extension through the diaphragm into the pleura or by lymphatic metastasis [11]. Positron emission tomography (PET) scanning can identify extra-peritoneal disease and nodal involvement not always visible on CT, and is therefore helpful in preoperative staging [9,13,14].

According to the World Health Organization, MPM is classified into three histological subtypes: epithelioid, sarcomatoid, and biphasic/mixed. This classification has both prognostic and therapeutic significance. The epithelioid subtype accounts for approximately 75% of cases and is associated with a more favourable prognosis, whereas the sarcomatoid subtype is the rarest and most aggressive; biphasic cases show both components [3,11,15,16]. The sarcomatoid subtype is variably cellular, composed of haphazardly arranged pleomorphic spindle cells within a fibrous stroma. Morphologically, epithelioid MPM resembles normal mesothelial cells, consisting of polygonal or cuboidal cells with eosinophilic or amphophilic cytoplasm, mild-to-moderate nuclear atypia, and variable mitoses. The architectural pattern is typically tubulopapillary, with admixed solid sheets surrounded by a desmoplastic stroma [3,15]. Differentiation from primary or metastatic carcinoma is essential, as these may have overlapping features [2,15].

Borderline mesothelial proliferations, including benign multicystic mesothelioma and well-differentiated papillary mesothelioma, have also been described [2,9]. These are distinct biological entities, differentiated from malignant counterparts by the absence of invasion, stromal cellularity, or complex papillary structures [2,3,15].

The diagnosis of mesothelioma is made on histological grounds and confirmed by a panel of immunohistochemical (IHC) stains. Mesothelial markers such as calretinin, D2-40, cytokeratin 5/6 (CK5/6), and Wilms tumour 1 (WT1) are typically positive, while epithelial markers such as carcinoembryonic antigen (CEA), Ber-EP4, MOC-31, and paired box gene 8 (PAX8) are negative, helping to distinguish MPM from peritoneal carcinoma or metastatic disease [3,10-12,14]. IHC can also detect loss of nuclear BAP1 expression. Homozygous deletion of BAP1 and cyclin-dependent kinase inhibitor 2A (CDKN2A/p16) by fluorescence in situ hybridisation (FISH) is highly specific for mesothelioma [5,11,13-15].

The management of MPM requires a multidisciplinary approach. The treatment algorithm is based on performance status, histologic type, and radiological assessment of resectability [16]. In selected patients with epithelioid disease, good performance status, and no metastatic spread, surgical tumour reduction (cytoreductive surgery, CRS) combined with hyperthermic intraperitoneal chemotherapy (HIPEC) remains the first-line treatment, with excellent improvements in survival reported [13,14]. Perioperative chemotherapy may be used in advanced or borderline-resectable cases, as well as in sarcomatoid or biphasic subtypes. For inoperable cases, systemic chemotherapy remains the mainstay of treatment [2,16]. Emerging therapies, including immunotherapy and targeted agents, are being explored as second-line options for progressive disease or chemotherapy intolerance [11,14]. In our case, the palliative route was pursued due to advanced disease and poor performance status.

This case also underscores the importance of integrating histopathological evaluation early in the diagnostic pathway when ascitic fluid cytology is inconclusive. Immunohistochemistry, including markers such as calretinin, WT1, and BAP1, plays a pivotal role in confirming the diagnosis and differentiating malignant peritoneal mesothelioma from metastatic adenocarcinoma. Such diagnostic precision not only guides appropriate management but also prevents unnecessary interventions in advanced disease.

This case highlights the challenges of diagnosing MPM in a patient presenting with abdominal distension, weight loss, and anorexia. The unusual features in this case included the absence of asbestos exposure, ascites initially attributed to IPMN, a serum-ascites albumin gradient (SAAG) pointing towards non-portal hypertension-related causes, cytology that was non-specific but reported as negative, and no definitive lesion on initial scans.

## Conclusions

Malignant peritoneal mesothelioma (MPM) is a rare condition that presents significant diagnostic challenges, often resulting in delayed diagnosis. While imaging can assist in identifying and characterising MPM, histological examination with immunohistochemistry remains the gold standard. Greater clinical awareness and a multidisciplinary approach are essential for timely diagnosis and effective management. Any patient with progressive abdominal distension and weight loss should raise suspicion for malignant ascites and prompt evaluation for an underlying peritoneal malignancy.

Histologic subtype is one of the most consistent predictors of survival. The epithelioid subtype is associated with a more favourable prognosis, whereas sarcomatoid and biphasic subtypes carry a poorer outlook. MPM was historically regarded as an end-stage disease with life expectancy often less than a year after diagnosis. However, recent therapeutic advances -- including systemic and peritoneal chemotherapy, immunotherapy, targeted molecular therapy, and combination approaches such as cytoreductive surgery with hyperthermic intraperitoneal chemotherapy (HIPEC) -- have shown promising outcomes in selected patients, though these were not applicable in our case due to advanced disease and poor performance status.
